# Impfquoten der Pneumokokken-Impfung bei Personen mit impfrelevanten Grunderkrankungen und Personen ab 60 Jahren – Eine Analyse von Sekundärdaten der Gesetzlichen Krankenversicherung (GKV)

**DOI:** 10.1055/a-2178-8306

**Published:** 2023-10-24

**Authors:** Sarah Mihm, Jörg Schelling, Regine Wölle, Andreas Suck, Dennis Häckl, Thomas Weinke, Timo Böllinger

**Affiliations:** 1 MSD SHARP & DOHME GmbH, München, Deutschland,; 2 LMU, Medizinische Fakultät, München, Deutschland,; 3 WIG2 GmbH, Leipzig, Deutschland,; 4 Universität Leipzig, Institut für öffentliche Finanzen und Public Management, Leipzig, Deutschland,; 5 Klinikum Ernst von Bergmann, Klinik für Gastroenterologie und Infektiologie, Potsdam, Deutschland,

**Keywords:** Pneumokokken-Erkrankungen, Routinedaten, Standardimpfung, Indikationsimpfung, Impfraten, pneumococcal diseases, insurance claims data, standard vaccination, indicated vaccination, vaccination rates

## Abstract

**Hintergrund** Die Ständige Impfkommission (STIKO) empfiehlt neben einer Standardimpfung für Personen ab 60 Jahren auch eine Indikationsimpfung gegen Pneumokokken, u.a. für Personen mit einer chronischen impfrelevanten Grunderkrankung – unabhängig vom Alter. In Deutschland werden durch das Robert-Koch Institut (RKI) regelmäßig Impfquoten für diese Gruppen publiziert, jedoch nicht nach zugrunde liegender Krankheit stratifiziert. Die Studie untersucht daher den Impfschutz von Personen mit chronischen Grundkrankheiten stratifiziert nach Krankheitsgruppen und -entitäten, die Rate von Wiederholungsimpfungen in dieser Population sowie die Impfquoten bei Personen ab 60 Jahren.

**Methoden** Der deskriptiven retrospektiven Kohortenstudie über den Zeitraum von 2014 bis 2019 liegt eine hinsichtlich Alter und Geschlecht für die deutsche Bevölkerung repräsentative Stichprobe des Instituts für angewandte Gesundheitsforschung (InGef) von etwa 4 Mio. GKV-Versicherten ab 16 Jahren zugrunde.

**Ergebnisse** Hochgerechnet auf die GKV-Grundgesamtheit betrug die Pneumokokken-Impfquote bei Personen ab 60 Jahren insgesamt 45,9% (von n= InGef-Standard-Impfkohorte: 1 009 763). Von allen Patienten mit chronischen Grunderkrankungen ab 16 Jahren besaßen nur 17,1% einen Impfschutz (von n= InGef-Indikationsimpfkohorte: 1 379 680). Stratifiziert nach Krankheitsentitäten wiesen Versicherte mit einem zugrunde liegenden Lungenemphysem mit 39,0% die höchste Impfquote auf (von n= 28 121). Von den Versicherten, die eine indizierte Erstimpfung erhalten haben, ließen sich nach 6 Jahren nur 23,9% erneut impfen (von n= InGef-Wiederholungsimpfkohorte: 12 328). Über alle Impfkohorten hinweg nahmen die Impfquoten mit zunehmendem Alter zu.

**Diskussion** Die Empfehlungen der STIKO zur Pneumokokken-Impfung bei Risikopatienten werden in Deutschland nicht in ausreichendem Maße umgesetzt. Obwohl die STIKO eine ausdrückliche Empfehlung bereits für Patienten mit 60 Jahren ausspricht, wies die Altersgruppe der 60- bis 64- Jährigen eine auffällig niedrige Impfquote auf (13,0% von n = 268 862). Darüber hinaus erhielt nicht einmal jede 5. Person ab 16 Jahren mit einer chronischen Grunderkrankung die empfohlene Indikationsimpfung. Um potenziellen Erkrankungen angemessen vorzubeugen, sollten höhere Impfraten angestrebt werden. Dies könnte vermutlich erzielt werden durch ein stringenteres Impfmanagement, geeignete Softwarelösungen mit Impferinnerung, monetäre Anreize für das Erreichen höherer Impfquoten sowie die Dokumentation des Impfstatus im Rahmen von Disease-Management-Programmen.

## Abstract (en)

**Background** In addition to standard vaccination for patients aged 60 and older, the Standing Committee on Vaccination (STIKO) recommends immunization against pneumococci for anyone at increased risk, including patients with chronic vaccine-relevant underlying conditions. In Germany, the Robert Koch Institute (RKI) regularly publishes vaccination coverage rates (VCRs) for these patient groups, without stratifying by other parameters. This study examines VCRs of patients with underlying chronic diseases, stratified by disease groups and entities, the re-vaccination rate, and VCRs in patients aged 60 years and older.

**Methods** This descriptive retrospective cohort study is based on a sample of about 4 million SHI-insured patients aged 16 years and older for the years 2014 to 2019, from the Institute for Applied Health Research (InGef) database. The sample is representative of age and sex distribution in the German population.

**Results** Extrapolated to the total SHI-population, the overall pneumococcal VCR in patients aged 60 years and older was 45.9 % (of n= InGef standard vaccination cohort: 1 009 763). Among all at-risk patients aged 16 years and older with chronic underlying diseases, only 17.1 % had received an indicated vaccination (InGef indicated vaccination cohort: 1 379 680). Stratified by disease entity, those with underlying pulmonary emphysema had the highest VCR, at 39.0 % (of n= 28 121). Of those who received a vaccination due to an underlying chronic disease, only 23.9 % were re-vaccinated after 6 years (InGef re-vaccination cohort: 12 328). Across all vaccination cohorts, VCRs increased with age.

**Discussion** The recommendations made by STIKO for pneumococcal vaccination based on age or an underlying chronic condition are not being implemented adequately in Germany. Although STIKO explicitly recommends vaccination from 60 years of age, the 60 to 64-year-old age group had a strikingly low VCR (13.0 % of n=268 862). Fewer than one in five patients aged 16 years and older with an underlying chronic condition had received the recommended indicated vaccination. To adequately prevent potential disease, higher vaccination rates should be targeted. This could probably be achieved through more stringent vaccination management, appropriate software solutions with vaccination reminders, monetary incentives for achieving higher vaccination rates and documentation of vaccination status in disease management programs.

## Hintergrund

Pneumokokken-Erkrankungen (Pneumococcal diseases – PD) sind symptomatische Infektionen, die durch das Bakterium *Streptococcus pneumoniae* verursacht werden. Zu den schwerwiegenden Pneumokokken-Erkrankungen, die unter dem Oberbegriff invasive Pneumokokken-Erkrankungen (Invasive Pneumococcal Diseases – IPD) zusammengefasst werden, gehören u.a. die Pneumokokken-Meningitis und die Bakteriämie 1 . Nach Angaben der Weltgesundheitsorganisation (WHO) sterben jedes Jahr 1,6 Millionen Menschen weltweit an PD 2 . Die Pneumokokken-Pneumonie ist die häufigste klinische Form der PD bei Erwachsenen. Die Sterblichkeitsrate liegt bei 5–7% und kann bei älteren Erwachsenen oder Personen mit bestimmten Grunderkrankungen wesentlich höher ausfallen 3 . Otitis media und Sinusitis sind häufige, aber weniger schwerwiegende Erscheinungsformen der PD. In den Industrieländern tragen Kinder im Alter von weniger als 2 Jahren und ältere Menschen die Hauptlast der Krankheit 2 .

In der Europäischen Union sind Impfstoffe zur Vorbeugung von PD als 2 Impfstoffarten erhältlich: zum Erhebungszeitpunkt der vorliegenden Studie als Pneumokokken-Polysaccharid-Impfstoff (PPSV), der 23 Serotypen abdeckt (PPSV23), und Pneumokokken-Konjugat-Impfstoffe (PCV), die 10 (PCV10) bzw. 13 Serotypen (PCV13) abdecken 4 . Nach Durchführung der Analyse fand im 2. Quartal 2022 als Folge der Zulassung durch die Europäische Arzneimittel-Agentur (EMA) der Launch von PCV15 5 und PCV20 6 statt, was sich nicht auf die Erkenntnisse der Analyse auswirkt, jedoch die Bedeutung der Impfstoffentwicklung aufzeigt. Die Ständige Impfkommission (STIKO) empfiehlt die Standardimpfung mit PPSV23 für alle Erwachsenen ab 60 Jahren. Für Kinder unter 2 Jahren wird eine PCV-Impfung in einem Schema von 2+1, also 3 Dosen, empfohlen 7 8 9 . Neben dem Alter stellt das Vorliegen von chronischen oder immunkompromittierenden Krankheiten einen weiteren impfrelevanten, altersunabhängigen Risikofaktor dar. Diesbezüglich können 2 Risikogruppen differenziert werden. Einerseits zählen Patienten, bei denen eine chronische Grunderkrankung wie Herzinsuffizienz oder Asthma bronchiale diagnostiziert wurde, zu der *„At-risk“* -Personengruppe, für die die Pneumokokken-Indikationsimpfung in Deutschland empfohlen wird 7 8 9 . Anderseits besteht die „ *High-risk“-* Personengruppe aus Patienten, bei denen eine immunkompromittierende Krankheit wie T-Zell-Defizienz oder eine HIV-Infektion diagnostiziert wurde und für die die Pneumokokken-Indikationsimpfung in Deutschland ebenfalls empfohlen wird 7 8 9 . Für die *„At-risk“* -Patienten wird entweder die sequenzielle Indikationsimpfung für Kinder und Jugendliche zwischen 2 und 15 Jahren oder ausschließlich der PPSV23-Impfstoff für Erwachsene ab 16 Jahren empfohlen. Für die *„High-risk“-* Patienten wird unabhängig vom Alter die sequenzielle Pneumokokken-Indikationsimpfung empfohlen, bei der zunächst ein 13-valenter Konjugat-Impfstoff (PCV13) genutzt wird, gefolgt von PPSV23 nach 6–12 Monaten. Aufgrund der begrenzten Dauer des Impfschutzes soll die Impfung mit PPSV23 in allen Risikogruppen mit einem Mindestabstand von 6 Jahren wiederholt werden 7 8 9 .

Um den Impfstatus der Bevölkerung zu ermitteln, führt das RKI im Rahmen des Projektes „KV-Impfsurveillance“ zur Überwachung (Surveillance) der Impfquoten eine bundesweite Statistik zu den Pneumokokken-Impfquoten 10 . Hierfür werden Abrechnungsdaten von allen 17 kassenärztlichen Vereinigungen (KVen) genutzt. Die zum Zeitpunkt der Studiendurchführung veröffentlichten Impfquoten beziehen sich auf die Standardimpfung für Erwachsene im Alter von 60–73 Jahren ohne impfrelevante Grunderkrankungen und auf Personen ab 18 Jahren mit impfrelevanten Grunderkrankungen 11 . Zielgruppenspezifische Impfdaten sind wichtige Voraussetzungen, um Trends, regionale Unterschiede und soziodemografische Besonderheiten beim Impfschutz darzustellen, aber auch zur Entwicklung gezielter Kommunikationsstrategien 10 . Trotzdem findet in der RKI-Statistik eine Stratifizierung der Impfquoten weder nach medizinischen Indikationen (bestimmten Krankheiten) noch regional nach Altersgruppen statt 11 .

Die vorliegende Studie schließt diese Forschungslücke und verfolgt 2 primäre Forschungsziele: die Ermittlung der Pneumokokken-Impfquoten einerseits für höhere Altersgruppen ab 60 Jahren und andererseits für Versicherte von mind. 16 Jahren mit chronischen, impfrelevanten Grunderkrankungen. Dabei werden die Auswertungen nach Krankheitsgruppen (bspw. chronische Herzkrankheiten) und -entitäten (bspw. Herzinsuffizienz) der Pneumokokken-Indikationsimpfung und der Pneumokokken-Wiederholungsimpfung stratifiziert. Als sekundäres Ziel der Studie gilt es außerdem festzustellen, ob es regionale Unterschiede bei den Pneumokokken-Impfquoten in den Studienpopulationen der primären Ziele gibt und durch welche ärztlichen Fachgruppen die Impfungen verabreicht wurden.

## Methoden

Für die retrospektive Analyse wurde die Forschungsdatenbank des Instituts für angewandte Gesundheitsforschung (InGef) genutzt. Diese Datenbank umfasst anonymisierte Krankenkassendaten von insgesamt 8,8 Millionen Versicherten, die in einer der datenliefernden Krankenkassen (vornehmlich Betriebs- und Innungskrankenkassen) versichert sind. Aus dieser Grundgesamtheit wird eine hinsichtlich Alter und Geschlecht für die deutsche Bevölkerung repräsentative Stichprobe im Umfang von 4,3 Millionen Versicherten gezogen 12 . Aus datenschutzrechtlichen Gründen konnte ein Zeitraum von 6 Jahren beobachtet werden, weshalb die vorliegende Studie den Zeitraum vom 1. Januar 2014 bis 31. Dezember 2019 umfasst.

Im Rahmen der Analyse wurde der Impfschutz sowohl bei der Standard-Pneumokokken-Impfung aufgrund fortgeschrittenen Alters über die Abrechnungsziffer 89119 der GKV als auch bei der Indikationspneumokokken-Erstimpfung über die Abrechnungsziffer 89120 und über die Abrechnungsziffer 89120R (bzw. 89119R 1 ) für die Wiederholungsimpfung betrachtet. Es wurden nur GKV-Versicherte in die Untersuchung einbezogen, die in der Betrachtungsperiode ununterbrochen bei einer der Krankenkassen versichert waren, welche Daten an die InGef-Datenbank weitergeleitet haben. Um die Vergleichbarkeit mit den Empfehlungen der STIKO zu den Risikogruppen zu ermöglichen und den Informationsgewinn durch die vorliegenden Ergebnisse zu den regelmäßig publizierten Pneumokokken-Impfquoten des RKI herauszustellen, wurden sowohl die Umsetzung der STIKO-Empfehlungen in der Schutzimpfungsrichtlinie (SI-RL) 9 als auch die Methodik des RKI in den epidemiologischen Bulletins 8 13 als Referenz für die Bildung der Analysepopulationen in der Stichprobe herangezogen. Eine ähnliche Vorgehensweise wurde für vergleichbare Studien bereits angewendet 14 .

Um die Pneumokokken-Impfquote im Jahr 2019 bei GKV-Versicherten ab 60 Jahren zu bestimmen, wurde eine 3-stufige Analyse durchgeführt. Zunächst wurde die Pneumokokken-Standardimpfquote von Versicherten im Alter von 60–65 Jahren im Jahr 2019 berechnet. Im 2. Schritt wurde die Impfquote im Jahr 2019 von Versicherten im Alter von 66 Jahren und älter, die zwischen 2014 und 2018 keine Impfung erhalten hatten, ausgewertet. Darauf basierend wurde die kumulative Pneumokokken-Standardimpfquote für Versicherte im Alter von 66 Jahren und älter im Jahr 2019 ermittelt. Da Versicherte im Alter ab 60 Jahren eine Standardimpfung (Abrechnungsziffer 89119), aber auch aufgrund des Vorliegens einer impfrelevanten Grundkrankheit eine Indikationsimpfung (Abrechnungsziffer 89120) erhalten können, wurden die Impfquoten für die beiden Abrechnungsziffern durch eine Sensitivitätsanalyse getrennt ermittelt.

Zur Bestimmung der Impfquote der Pneumokokken-Indikationsimpfung wurden GKV-Versicherte im Alter von mindestens 16 Jahren mit einer impfrelevanten chronischen Grunderkrankung (d.h. die *„At-risk“* -Personengruppe) anhand der ICD-10-GM-Codes im Jahr 2019 aufgegriffen. Die Versicherten mussten hierbei mindestens 2 ambulante Diagnosen in 2 verschiedenen Quartalen oder mindestens 1 stationäre Diagnose einer bestimmten Krankheitsgruppe oder Krankheitsentität im Jahr 2019 aufweisen. Darauf basierend wurde der Impfschutz für diese Personengruppe für die 6 Jahre von 2014–2019 ausgewertet. Schließlich wurden die Impfquoten nach 4 Krankheitsgruppen, 9 Krankheitsentitäten sowie 8 Altersgruppen stratifiziert.

In einem 3-stufigen Verfahren wurde die Impfquote der Pneumokokken-Wiederholungsimpfung ermittelt. Zuerst wurden GKV-Versicherte, die 2019 das 16. Lebensjahr vollendet und an einer chronischen, impfrelevanten Grunderkrankung gelitten haben, herangezogen. Beim Aufgriff der Versicherten wurden dieselben Diagnosekriterien wie beim Aufgriff der Versicherten für die Indikationsimpfung genutzt. Hiervon wurden diejenigen selektiert, die 2012 oder 2013 eine Indikationsimpfung hatten; da die Jahre 2012 und 2013 nur bezüglich des Vorliegens einer Impfung herangezogen wurden, durften diese im Rahmen einer datenschutzrechtlichen Ausnahme genutzt werden. Da die STIKO Wiederholungsimpfungen im Abstand von mindestens 6 Jahren empfiehlt, wurde der Zeitraum von 2018–2019 als Auswertungszeitraum für die Bewertung der Wiederholungsimpfungen herangezogen. Dies reduziert das Risiko, dass die in der Regel innerhalb von 6–12 Monaten nach der Erstimpfung folgende, sequenzielle Impfung bei Menschen mit Immunschwäche oder unter Immunsuppressiva fälschlicherweise als Wiederholungsimpfung klassifiziert wird.

Die deskriptiven Auswertungen wurden als relative Häufigkeiten in den jeweiligen Impfkohorten ausgewiesen. Alle Werte sind hochgerechnet auf die GKV.

## Ergebnisse

**Abb. 1:**
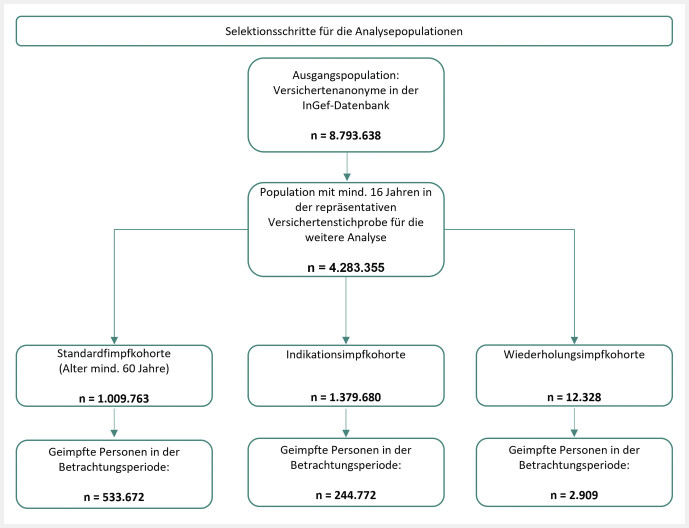
Übersicht der Selektionsschritte für die Analysepopulationen der jeweiligen Impfkohorten.

Insgesamt umfasste die Standardimpfkohorte 100 9763 GKV-Versicherte, die Indikationsimpfkohorte 1 379 680 und die Wiederholungsimpfkohorte 12 328. Die Population der Standardimpfung wurde nach Altersgruppe (60–64, 65–69, 70–74, 75+) und Bundesland stratifiziert, wohingegen die Indikations- und Wiederholungsimpfkohorten nach Altersgruppe (16–29, 30–39, 40–49, 50–59, 60–64, 65–69, 70–74, 75+), nach spezifischer Krankheitsgruppe und Krankheitsentität sowie nach Bundesland differenziert betrachtet wurden. Die nachfolgende Abb. 1 zeigt eine Übersicht zu den Analysepopulationen der jeweiligen Impfkohorten. Die anschließenden Abschnitte enthalten detaillierte Erläuterungen zu den wichtigsten Erkenntnissen, bezogen auf die verschiedenen Impfkohorten.

### Impfschutz der GKV-Versicherten ab 60 Jahren

**Abb. 2:**
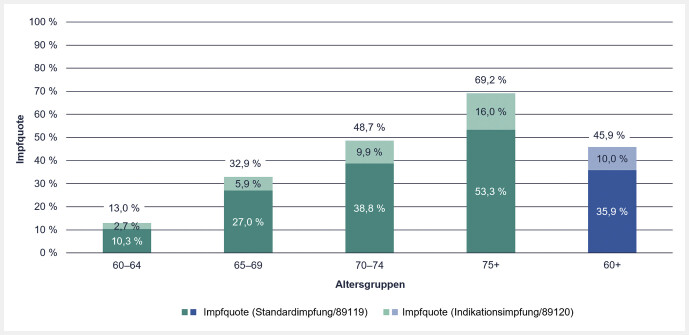
Impfquote der Pneumokokken-Impfung bei Versicherten im Alter von mindestens 60 Jahren, stratifiziert nach Altersgruppe und Abrechnungsziffer für die Standard- bzw. Indikationsimpfung.

Die Pneumokokken-Impfquote bei Versicherten im Alter von mindestens 60 Jahren betrug im Jahr 2019 insgesamt 45,9% von n= InGef-Standard-Impfkohorte: 1 009 763. Bei einer differenzierten Betrachtung je Altersgruppe, wie in Abb. 2 dargestellt, zeigt sich ein Anstieg der Impfquote mit zunehmendem Alter: Versicherte in der Altersgruppe der 60- bis 64-Jährigen hatten die geringste Impfquote (13%), während Versicherte im Alter von mindestens 75 Jahren mit 69,2% die höchste Impfquote aufwiesen.

Da Personen im Alter von 60 Jahren und älter sowohl aufgrund ihres Alters als auch aufgrund einer Indikation eine Impfung erhalten können, wurde die Impfquote im Rahmen einer Sensitivitätsanalyse nach der genutzten Abrechnungsziffer stratifiziert. Diese Analyse zeigte, dass die altersbedingte Standardimpfung (Abrechnungsziffer 89119) zu einer Impfquote von 35,9% führte, während 10% der Impfungen aufgrund einer medizinischen Indikation erfolgten (Abrechnungsziffer 89120). Beide Impfquoten nehmen mit steigendem Alter zu.

**Abb. 3:**
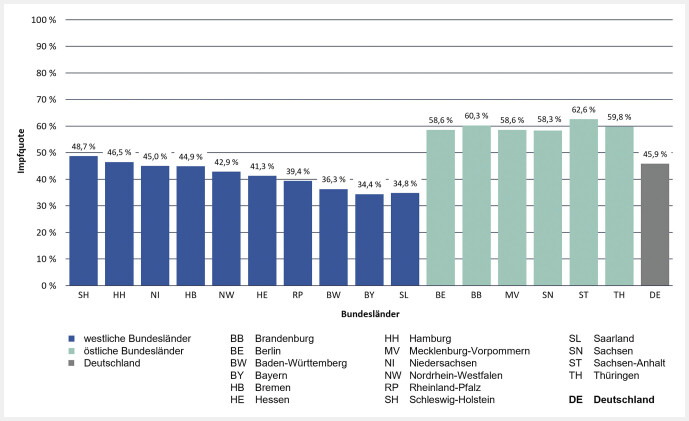
Impfquote der Pneumokokken-Impfung bei Versicherten im Alter von mindestens 60 Jahren, stratifiziert nach Bundesland.

Bei einer regionalen Betrachtung der Impfquoten unter Personen ab 60 Jahren zeigt sich ein höherer Impfschutz bei Versicherten in östlichen Bundesländern (inkl. Berlin), verglichen mit denen in westlichen Bundesländern ( Abb. 3 ). So war altersgruppenübergreifend betrachtet die Impfquote in Bayern am niedrigsten (34,4%) und in Sachsen-Anhalt am höchsten (62,6%). Dieser Trend zeigte sich auch bei den Altersgruppen von 60–64 Jahren (7,7% gegenüber 23,9%), von 65–69 Jahren (22,1% gegenüber 50,8%) und von 70–74 Jahren (35,3% gegenüber 68,3%). Bei Versicherten im Alter von mindestens 75 Jahren hatte ebenfalls Sachsen-Anhalt mit 86,11% die höchste Impfquote – und das Saarland (55,2%) sowie Bayern (55,3%) hatten die niedrigsten Impfquoten.

### Impfschutz der GKV-Versicherten ab 16 Jahren mit chronischen Grunderkrankungen

Die Impfquoten der Pneumokokken-Indikationsimpfung für GKV-Versicherte ab 16 Jahren mit n= InGef-Indikationsimpfkohorte: 1 379 680 wurden stratifiziert nach Krankheitsgruppe und -entität, hier in Tab. 1 dargestellt. Der Aufgriff und die Einteilung der Indikationen orientierten sich am Klassifikationsmodell für den Risikostrukturausgleich (Ausgleichsjahr 2020), die ICD-Codes wurden in einem ersten Schritt den Diagnosegruppen, daraufhin hierarchisierten Morbiditätsgruppen und schließlich Krankheitsgruppen zugeordnet 15 .

**Table:** **Tab. 1** Impfquote der Pneumokokken-Indikationsimpfung bei Versicherten mit chronischen, impfrelevanten Erkrankungen im Alter von mindestens 16 Jahren, stratifiziert nach Krankheitsgruppe und nach Krankheitsentität.

Krankheitsgruppen aufgeschlüsselt nach Krankheitsentitäten	Impfquote GKV in %
Chronische Herzkrankheiten, darunter explizit ausgewertet:	24,1
Herzinsuffizienz	26,7
Koronare Herzkrankheit	26,7
Herzklappenerkrankung	27,6
Vorhofflimmern	25
Chronische Erkrankungen der Atemwege, darunter explizit ausgewertet:	21,5
Asthma	17,6
Chronisch Obstruktive Lungenerkrankung (COPD)	31,5
Lungenemphysem	39
Stoffwechselkrankheiten, darunter explizit ausgewertet:	18,5
Diabetes mellitus, Typ I und II	24,3
Neurologische Erkrankungen, darunter explizit ausgewertet:	19
Epilepsie	14,2
Zerebralparese	9,2
**Jegliche Indikationskrankheit**	**17,1**

Nach Krankheitsgruppen stratifiziert, war die Impfquote bei Versicherten mit chronischen Herzerkrankungen im Jahr 2019 am höchsten (24,1%), gefolgt von Versicherten mit chronischen Erkrankungen der Atemwege (21,5%) und mit neurologischen Erkrankungen (19%). Am niedrigsten war die Impfquote hingegen für die Krankheitsgruppe der Stoffwechsel-Erkrankungen (18,5%).

Stratifiziert nach Krankheitsentitäten wiesen Versicherte ab 16 Jahren mit einem zugrunde liegenden Lungenemphysem mit 39% die höchste Impfquote auf, gefolgt von Versicherten mit chronisch obstruktiver Lungenerkrankung (31,5%). Unter den untersuchten Krankheitsentitäten war die Impfquote bei Versicherten mit einer zugrunde liegenden Zerebralparese am niedrigsten (9,2%). Insgesamt hatten nur 17,1% der GKV-Versicherten ab 16 Jahren, die als Risikopatienten galten, eine Impfung wegen einer zugrunde liegenden chronischen Indikationskrankheit verabreicht bekommen.

**Abb. 4:**
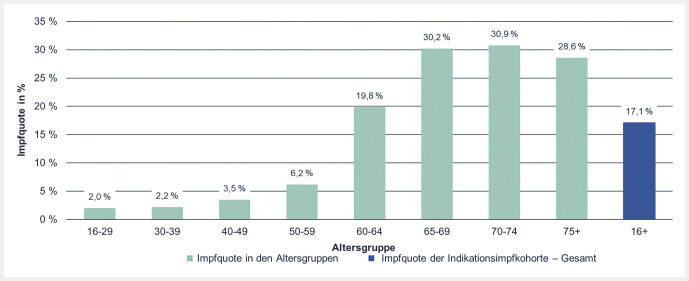
Impfquote der Pneumokokken-Indikationsimpfung bei Versicherten mit chronischen, impfrelevanten Erkrankungen im Alter von mindestens 16 Jahren, stratifiziert nach Altersgruppe.

**Abb. 5:**
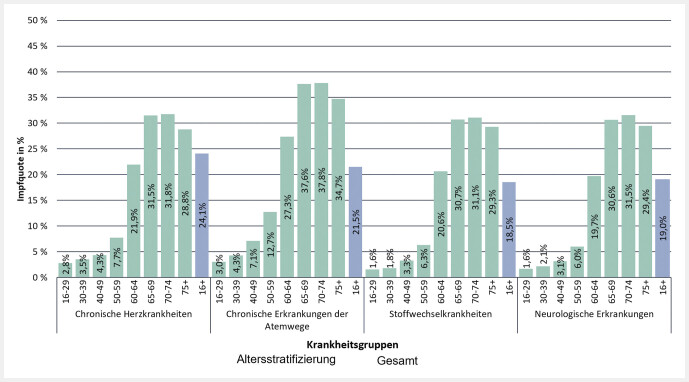
Impfquote der Pneumokokken-Indikationsimpfung bei Versicherten mit chronischen, impfrelevanten Erkrankungen im Alter von mindestens 16 Jahren, stratifiziert nach Krankheits- und Altersgruppen

Die Pneumokokken-Impfquoten für Versicherte ab 16 Jahren mit einer chronischen Krankheit, für die eine Pneumokokken-Impfung empfohlen wird, wurden wiederum nach Altersgruppen stratifiziert ( Abb. 4 und Abb. 5 ). Insgesamt war die Impfquote bei älteren Versicherten höher als bei jüngeren und stieg mit zunehmendem Alter an. Die höchste Impfquote wiesen Versicherte in der Altersgruppe der 70- bis 74-Jährigen (30,9%) auf, gefolgt von Versicherten im Alter zwischen 65 und 69 Jahren (30,2%). Zwischen den Altersgruppen der 50- bis 59-Jährigen und der 60- bis 64-Jährigen wurde ein deutlicher Sprung der Pneumokokken-Indikationsimpfquote bei chronisch Erkrankten von 6,2% auf 19,8% beobachtet. Ein weiterer steiler Anstieg folgte zu der Personengruppe im Alter zwischen 65 und 69 Jahren auf 30,2%. Alle Altersgruppen unter 60 Jahren wiesen eine Pneumokokken-Impfquote von weniger als 10% auf.

In der gemeinsamen Betrachtung von Alters- und Krankheitsgruppen zeigte sich, dass die 16- bis 29-Jährigen den geringsten Impfschutz hatten. Die Impfquote lag hier bei 1,6% für Stoffwechselkrankheiten, 1,6% für neurologische Erkrankungen, 2,8% für chronische Herzkrankheiten und 3% für chronische Erkrankungen der Atemwege. In der Altersgruppe der 70- bis 74-Jährigen waren die Impfquoten – wenn man alle Altersgruppen betrachtet – bei allen 4 Krankheitsgruppen am höchsten: 31,1% für Stoffwechselkrankheiten, 31,5% für neurologische Krankheiten, 31,8% für chronische Herzkrankheiten und 37,8% für chronische Erkrankungen der Atemwege.

### Wiederholungsimpfquote nach indizierter Erstimpfung bei chronischen Krankheiten

Die Pneumokokken-Wiederholungsimpfquoten bei GKV-Versicherten ab 16 Jahren mit einer chronischen, impfrelevanten Grunderkrankung mit n= InGef-Wiederholungsimpfkohorte: 12 328 sind, stratifiziert nach Altersgruppen, in Tab. 2 zusammengefasst. Insgesamt lag die Wiederholungsimpfquote bundesweit bei 23,9%, d.h. weniger als jede 4. geimpfte Person mit einer Risikokrankheit erhielt nach 6 Jahren die von der STIKO empfohlene Wiederholungsimpfung. Die Impfquote war bei Versicherten im Alter von 16–29 Jahren am niedrigsten (9,7%) und stieg mit zunehmendem Alter an. Die insgesamt höchste Impfquote mit 28,1% wurde in der Altersgruppe der 65- bis 69-Jährigen festgestellt. Diese Altersgruppe hatte auch in allen 4 Krankheitsgruppen den höchsten Impfschutz.

**Table:** **Tab. 2** Impfquoten der Pneumokokken-Wiederholungsimpfungen bei Versicherten im Alter von mindestens 16 Jahren, stratifiziert nach Altersgruppen.

Altersgruppe	Impfquote GKV in %
16–29	9,7
30–39	16,5
40–49	15,9
50–59	22,7
60–64	26,6
65–69	28,1
70–74	24,2
75+	25,2
**16+**	**23,9**

### Die impfenden Ärzte

Die Analyse zeigte, dass der Großteil der Impfungen sowohl in der Standard-Impfkohorte (Versicherte ab 60 Jahren) als auch in den Indikations- und Wiederholungsimpfkohorten (Versicherte ab 16 Jahren mit chronischen Grunderkrankungen) von Allgemeinärzten verabreicht wurden. Die durchschnittliche Wahrscheinlichkeit, dass ein Allgemeinmediziner die Impfung in der Standard-Impfkohorte verabreichte, lag bundesweit bei 95,2% [95%-Konfidenzintervall: 95%; 95,3%]; sie war mit 98,3% in Sachsen am höchsten und mit 88,7% in Hamburg am niedrigsten. Für die Indikations- und Wiederholungsimpfungen lag dieselbe Wahrscheinlichkeit bei 94,4% bzw. 93,1%. Unter den Fachärzten haben die Pneumologen mit einer Wahrscheinlichkeit von jeweils 1,5%, 2,3% und 3,1% den Impfstoff in den Standard-, Indikations- und Wiederholungsimpfkohorten am häufigsten verabreicht.

## Diskussion

Die Ergebnisse der vorliegenden Arbeit stellen eine wertvolle Ergänzung zu der RKI-Statistik dar, die ausschließlich die Pneumokokken-Standardimpfquoten für Erwachsene im Alter von 60–73 Jahren ohne Grunderkrankungen und für Personen ab 18 Jahren mit einer impfrelevanten Grunderkrankung aufführt 11 . Die Studie konnte unseres Wissens erstmals Impfquoten der Pneumokokken-Indikationsimpfung bei Versicherten im Alter von mindestens 16 Jahren, stratifiziert nach zugrunde liegender, impfrelevanter, chronischer Krankheit darstellen. Trotz der ausdrücklichen Empfehlungen der STIKO zur Pneumokokken-Impfung bei Risikokrankheiten fiel die Impfquote der *„At-risk“* -Personengruppe vorliegend sehr niedrig aus. Nur 17,1% der Patienten ab 16 Jahren mit einer für die Pneumokokken-Impfung relevanten, chronischen Risikoindikation waren im Jahr 2019 geimpft. Bei 2 der betrachteten Krankheitsgruppen hatte nicht einmal jeder 5. Patient einen Impfschutz: Die Impfquote unter Patienten mit Stoffwechsel-Erkrankungen betrug 18,5%, während Risikopersonen mit neurologischen Erkrankungen eine Impfquote von 19% aufwiesen. Auffällig gering waren zudem die Impfquoten bei allen Altersgruppen der *„At-risk“-* Patienten unter 60 Jahren, über alle Krankheitsgruppen hinweg: Die Impfquoten lagen hier jeweils unter 10% und mit 2% war die Impfquote bei den 16- bis 29-Jährigen am niedrigsten. Dies könnte ein Hinweis darauf sein, dass das Alter als zusätzliche Indikation, neben den schon impfrelevanten chronischen Erkrankungen, von den Ärzten für die Verabreichung des Impfstoffs empfunden wird. Aufgeschlüsselt nach den einzelnen Krankheitsbildern stellte sich heraus, dass Patienten ab 16 Jahren mit Zerebralparese insgesamt den niedrigsten Impfschutz (9,2%) hatten, während 39% der von einem Lungenemphysem Betroffenen eine Pneumokokken-Impfung bekamen.

Wertvolle zusätzliche Informationen liefert auch die Sensitivitätsanalyse für Versicherte ab 60 Jahren, unabhängig von ihrem Gesundheitszustand, die die getrennte Betrachtung der Standard- und Indikationsimpfungen für diese Risikogruppe ermöglicht. Diesbezüglich ergab die vorliegende Analyse, dass die Impfquote bei Personen ab 60 Jahre im Jahr 2019 insgesamt 45,9% betrug, wobei 35,9% auf das Alter und 10% auf eine chronische Indikationskrankheit zurückzuführen waren. Dank der Stratifizierung der Standardimpfkohorte nach Altersgruppen und der Erweiterung der Studienpopulation auf Personen, die älter als 75 Jahre sind, konnten nun weitere Einblicke in das Impfgeschehen gewonnen werden. So zeigte sich, dass die Impfquoten mit zunehmendem Alter anstiegen und in der Altersgruppe der über 75-Jährigen am höchsten waren. Obwohl die STIKO-Standardempfehlung für die Pneumokokken-Impfung bei 60 Jahren beginnt, wies die Altersgruppe der 60- bis 64-Jährigen mit 13% eine auffällig niedrige Impfquote auf.

Die STIKO empfiehlt eine Wiederholungsimpfung nach mindestens 6 Jahren für Risikopatienten ab 16 Jahren aufgrund vorliegender, impfrelevanter Krankheiten. Unsere Analyse zeigte zum ersten Mal, dass die Rate der Wiederholungsimpfungen bei *„At-risk“-* Personen mit mindestens 16 Jahren in Deutschland im Betrachtungszeitraum bei 23,9% lag, d.h. weniger als jeder 4. geimpfte Risikopatient erhielt nach einer indizierten Erstimpfung die von der STIKO empfohlene Wiederholungsimpfung. Die Analyse zeigte zudem, dass die Pneumokokken-Impfung fast ausschließlich von den Hausärzten verabreicht wurde.

Die Ergebnisse der Studie müssen allerdings auch im Kontext von Limitationen betrachtet werden, die weitgehend auf das Studiendesign mit Routinedaten der GKV sowie den durch den Datenschutz eingeschränkten Beobachtungszeitraum zurückzuführen sind. Zunächst könnte die Verwendung von Abrechnungsdaten, die per definitionem nicht zu Forschungszwecken erhoben wurden, sondern im Rahmen der Routineversorgung entstanden sind, die Repräsentativität der Studienergebnisse wegen Verzerrungen und des Confoundings (Störfaktoren) einschränken. Nichtsdestotrotz ist insgesamt von einer guten Gesamtrepräsentativität der InGef-Forschungsdatenbank für die deutsche Bevölkerung in Bezug auf Alter, Geschlecht, Morbidität, Mortalität und Arzneimittelverbrauch auszugehen, wie es frühere Studien nachweisen konnten 16 . Da vor allem Betriebs- und Innungskrankenkassen ihre Abrechnungsdaten in die InGef-Datenbank einfließen lassen, können Unterschiede zwischen der Datenbankpopulation und der allgemeinen deutschen Bevölkerung in Bezug auf den sozioökonomischen Status, das Gesundheitsbewusstsein, die Inanspruchnahme von Vorsorgeleistungen und den Beschäftigungsstatus allerdings nicht ausgeschlossen werden. Aufgrund der deutschen Datenschutzbestimmungen wurde der Beobachtungszeitraum auf 6 Jahre (2014–2019) eingegrenzt. Dies könnte dazu geführt haben, dass die Impfhistorie – insbesondere bei älteren Versicherten – nicht genau abgebildet werden konnte: Falls die Impfraten vor 2014 signifikant höher oder niedriger waren als zwischen den Jahren 2014 und 2019, könnten die Impfraten für Versicherte, die vor 2014 60 Jahre oder älter waren, unter- bzw. überschätzt werden. Eine gewisse Unterschätzung der Wiederholungsimpfquote ist unter dem Studiendesign ebenfalls denkbar. Die STIKO empfiehlt für Risikopatienten eine Wiederholungsimpfung nach mindestens 6 Jahren. Vorliegend wurden Patienten mit einer Erstimpfung im Jahr 2012 oder 2013 eingeschlossen und die Jahre 2018 und 2019 für die Wiederholungsimpfung ausgewertet. Wenn Ärzte die Impfung erst nach 8 Jahren oder später verabreicht haben, entspricht dies zwar den STIKO-Empfehlungen, jedoch blieben diese Impfungen in der vorliegenden Analyse unberücksichtigt. Schließlich interessierten uns im Zuge unserer Untersuchungen die Pneumokokken-Indikationsimpfquoten von Risikopatienten insgesamt. Aus diesem Grund wurden die *„High-risk“-* Patienten aus der Indikationsimpfkohorte der *„At-risk“-* Personen nicht ausgeschlossen. Dies könnte zu höheren Impfquoten in der Indikationsimpfkohorte geführt haben. Allerdings schätzen wir dies als eine unerhebliche Einschränkung der Studie ein, da frühere Untersuchungen gezeigt haben, dass nur 4,4% aller Patienten mit einer immunkompromittierenden Erkrankung innerhalb von 2 Jahren nach der ersten Diagnose gegen Pneumokokken geimpft wurden 17 .

Anhand der Studienergebnisse lässt sich feststellen, dass die Empfehlungen der STIKO zur Pneumokokken-Impfung bei Patienten, die aufgrund des Alters oder aufgrund einer chronischen, impfrelevanten Grunderkrankung besonders gefährdet sind, in Deutschland nicht in ausreichendem Maße umgesetzt werden, was auch neueste Erhebungen zeigen. In Anbetracht der alternden Gesellschaft und der Prävalenzzunahme chronischer Krankheiten stellt dies einen besorgniserregenden Zustand dar. Um potenziellen Erkrankungen angemessen vorzubeugen, sollte das Bewusstsein für die Krankheitslast durch Pneumokokken und die Bedeutung von Schutzimpfungsprogrammen – sowohl bei Patienten als auch bei den behandelnden Ärzten – geschärft werden. Höhere Impfraten könnten vermutlich durch ein stringenteres Impfmanagement in den Praxen erreicht werden 18 . Durch geeignete Softwarelösungen würden Ärzte an durchzuführende Impfungen erinnert und es könnte darüber auch der Lagerbestand kontrolliert werden. Hierbei wäre überlegenswert, inwiefern das Impfmanagement in den Praxen durch monetäre Anreize intensiviert werden könnte. Unabhängig hiervon könnte bei der Gestaltung von Disease-Management-Programmen die Dokumentation des Impfstatus erwogen werden, was sowohl bei Patienten als auch bei Ärzten die Bedeutung des Impfstatus in der Wahrnehmung erhöhen würde.

Mit den Erkenntnissen der vorliegenden Analyse können einzelne, vulnerable Zielgruppen gezielt angesprochen werden, wodurch der Erfolg der Impfkampagne maßgeblich unterstützt wird.

**Kernaussagen**

- Die Pneumokokken-Impfquote bei Personen ab 60 Jahren betrug insgesamt 45,9%.
- Von allen Patienten mit chronischen Grunderkrankungen ab 16 Jahren besaßen nur 17,1% einen Impfschutz.
- Stratifiziert nach Krankheitsentitäten wiesen Versicherte mit einem zugrunde liegenden Lungenemphysem mit 39,0% die höchste Impfquote auf.
- Von den Versicherten, die eine indizierte Erstimpfung erhalten haben, ließen sich nach 6 Jahren nur 23,9% erneut impfen.
- Über alle Impfkohorten hinweg nahmen die Impfquoten mit zunehmendem Alter zu.
- Es zeigten sich regionale Unterschiede im Impfschutz der Populationen: Die Versichertengruppen in den östlichen Bundesländern wiesen tendenziell höhere Impfquoten im Vergleich zu denen in den westlichen Bundesländern auf.

**Addendum**

Die Empfehlung der Ständigen Impfkommission für die Pneumokokken-Impfung Erwachsene wurde nach Annahme des Manuskripts und vor Druck aktualisiert. Die STIKO empfiehlt nun den Pneumokokken-Impfstoff PCV20 für Erwachsene. Die neue Empfehlung und Wissenschaftliche Begründung sind im Epidemiologischen Bulletin 39/2023 veröffentlicht. An den definierten Indikationsgruppen für eine Pneumokokken-Impfung wurden keine Änderungen vorgenommen.

## Footnotes

1
Laut G-BA ist für die Auffrischungsimpfung von Risikopatienten nur 89120R zu kodieren 19 . Um Kodierfehler auszuschließen, wird in der Analyse jedoch auch der Code 89119R verwendet.
